# Integrated Metabolomic and Transcriptomic Analysis Reveals the Molecular Regulatory Mechanism of Gamma-Aminobutyric Acid Accumulation in White Quinoa (*Chenopodium quinoa* Willd.) in Response to Dark and Ultrasound Stress

**DOI:** 10.3390/foods14071186

**Published:** 2025-03-28

**Authors:** Mengying Wu, Qian Zhou, Yasai Sun, Liangfu Zhou, Dongyao Li, Ting Ren, Yu Zheng, Wen Zhao, Jie Wang

**Affiliations:** College of Food Science and Technology, Hebei Agricultural University, Baoding 071001, China; wumy9902@163.com (M.W.); zhouqian@hebau.edu.cn (Q.Z.); sunyasai@hebau.edu.cn (Y.S.); zhoulf202201@163.com (L.Z.); lidongyao@hebau.edu.cn (D.L.); rt1134771972@163.com (T.R.); zhengyu9818@163.com (Y.Z.)

**Keywords:** *Chenopodium quinoa* Willd., γ-aminobutyric acid, abiotic stress, ultrasound, metabolome, transcriptome

## Abstract

Gamma-aminobutyric acid (GABA) is a nonprotein amino acid, which confers stress resistance to plants. Precise mechanisms underlying GABA accumulation in quinoa (*Chenopodium quinoa*) subjected to dark and ultrasonic stresses have not been elucidated. We conducted transcriptome and metabolome analyses of quinoa samples exposed to various stress treatments to reveal molecular pathways leading to GABA accumulation. Through the comprehensive integration of metabolome and transcriptome data, an association was revealed between GABA accumulation, 9 differentially expressed metabolites, and 27 differentially expressed genes. Two pathways responsible for GABA synthesis were identified, involving glutamate decarboxylase and aldehyde dehydrogenases, respectively. These enzymes regulate the enrichment of GABA in quinoa under dark and ultrasonic stress conditions. We demonstrated that under ultrasonic stress, proline and alanine increased, whereas glutamate and arginine declined. Phenolic acid, flavonoids, and alkaloid metabolites increased. These findings provide novel insights into the mechanism by which darkness and ultrasound stress enhance GABA, supporting the development of targeted synthetic biology techniques.

## 1. Introduction

Gamma-aminobutyric acid (GABA) is a four-carbon nonprotein amino acid characterized by its linkage to a carbon atom, positioned two carbons away from the carboxyl group, through an amino group. Serving as a pivotal inhibitory neurotransmitter within the intricate animal nervous system, GABA assumes a critical role in orchestrating the synaptic integration of newly formed neurons within the mature brain [1] and in modulating the activation of the hypothalamic–pituitary–adrenal axis [2]. Beyond its neural functions, GABA is renowned for its diverse array of health-promoting attributes, encompassing neuroprotective, antidepressant, anti-insomniac, antidiabetic, antihypertensive, and anti-inflammatory properties [3,4,5,6,7]. Notably, the monograph issued by Canada’s Natural and Non-Prescription Health Products Directorate for Cognitive Function Products, alongside the recommendations from global manufacturers, advocate for a daily GABA intake ranging from 50 mg to 3000 mg and 1.5 µg to 3000 mg, respectively [8]. Despite these endorsements, the inherent GABA content in dietary sources tends to be modest. Consequently, the enhancement of GABA levels in food products through safe physical methodologies emerges as a promising strategy, garnering greater favor among health-conscious consumers, in contrast to supplementation with external sources.

The process of germination holds the potential to enhance the nutritional profile of whole grains significantly. By instigating the activity of pertinent enzymes and hormones, appropriate germination conditions involving factors such as temperature, light exposure, and moisture levels can catalyze the transition of grains from a dormant state to one of active metabolic processes, thus facilitating the liberation of essential nutrients [9]. Dark stress exerts a notable influence on the energy metabolism of plants, culminating in the accumulation of organic acids within the tricarboxylic acid (TCA) cycle and alterations in the amino acid composition in rice [10]. Noteworthy as a signaling mediator, GABA undergoes substantial accumulation in plants confronted with abiotic stresses, thereby bolstering their resilience against such adversities [11]. Ultrasound stress has garnered escalating interest as a non-thermal technique in the processing of plant-derived foods, as it amplifies the enrichment of primary and secondary metabolites [12]. Following ultrasonic treatment (25 kHz, 5 min), germinated wheat and red rice exhibit augmented levels of beneficial and flavorful plant metabolites, including riboflavin, GABA, glucose, and free sugars [13]. Moreover, ultrasonic exposure of 5 min leads to heightened concentrations of GABA, alanine, succinic acid, total serotonin, and total polyphenols in sprouted oats [14]. The application of ultrasound facilitates amylolysis in whole-grain brown rice, elevating the levels of reducing sugars, GABA, proline, and antioxidants [15]. The impact of ultrasound-assisted germination on the abundance of functional phytochemicals is purportedly intertwined with the plants’ adaptive response to diverse stressors [13], ultimately shaping their phenotypic traits, nutritional attributes, and functional characteristics.

Common abiotic stress techniques include low-temperature, salt, and hypoxia treatments, which have disadvantages, such as excessive salt accumulation, the necessity for sealed instruments, and low efficiency. In contrast, ultrasonic treatment is recognized for its environmental sustainability, as it does not introduce foreign chemicals [16]. This method not only enhances the concentration of GABA, phenolic compounds, and nutritional value but also contributes to reducing microbial load and eliminating pollutants from the product [12]. Nonetheless, the intricate mechanism behind GABA enrichment under ultrasonic stress remains insufficiently explored. Given that GABA is prevalently found in modest quantities across various plant species, which often fall short of meeting the physiological requirements of the human body [17], delving into the mechanisms orchestrating GABA enrichment could yield valuable insights for enhancing its production efficiency via synthetic biology methodologies. Notably, GABA assumes a pivotal role in modulating the metabolic pathways of carbon and nitrogen by establishing a link between amino acid metabolism and the TCA cycle [18]. In plants, the conversion of glutamate to GABA is catalyzed by the enzyme glutamate decarboxylase (GAD), leading to the production of succinate semialdehyde through the subsequent action of GABA transcarboxylase (GABA-T) within the mitochondria. The succinate semialdehyde is further metabolized into succinate via succinate semialdehyde dehydrogenase (SSADH), eventually integrating into the TCA cycle. Although GABA conventionally undergoes synthesis via the GABA shunt pathway, its production can also be instigated under stress conditions through the breakdown of specific polyamines, such as spermine, spermidine, putrescine, and proline [19,20,21]. For instance, during barley germination, the conversion of spermine into GABA under ultraviolet B (UV-B) light stress contributes to alleviating UV-B-induced oxidative harm [22]. Similarly, in tea plants, the genes *CsAMADH1* within the plastids and *CsCuAO1* and *CsCuAO3* within the peroxisomes mediate the transformation of putrescine into GABA [23].

Quinoa stands out as the sole grain acknowledged by the Food and Agriculture Organization capable of meeting all human nutritional requirements in a single crop. Endowed with gluten-free properties, a comprehensive array of essential amino acids, fatty acids, and a bounty of phytochemicals, such as saponins and polyphenols [24], quinoa has garnered favor among consumers. Our antecedent investigations have unveiled the potential of ultrasound and dark stress in augmenting GABA levels during the germination of quinoa [25]. Nevertheless, the underlying mechanism governing this enhancement remains unclear.

Hence, the present study analyzed the metabolomes and transcriptomes of quinoa samples subjected to dark and ultrasound stress to unravel the intricate molecular pathways underpinning GABA accumulation in quinoa. The primary objective was to unveil potential rate-limiting genes in the GABA metabolic pathway. The results of this study will contribute to an in-depth understanding of the molecular mechanisms by which ultrasound and dark stress influence the enrichment of GABA in quinoa. Furthermore, they hold promise for informing the advancement of ultrasound and dark stress combined technology, aimed at developing innovative quinoa processing techniques for the production of functional whole-grain foods.

## 2. Materials and Methods

### 2.1. Quinoa Cultivation and Ultrasonic Treatment

The Jili 3 cultivar of quinoa was cultivated and harvested at the Zhangjiakou Yuerwan Quinoa Planting Cooperative (Zhangjiakou City, China). GABA (99%), amino acid standards (98%), and chromatographically pure acetonitrile were purchased from Sigma-Aldrich (Shanghai, China), Shanghai Yuanye Bio-Technology Co., Ltd. (Shanghai, China), and Macklin Biochemical Co., Ltd. (Shanghai, China), respectively.

**Figure 1:**
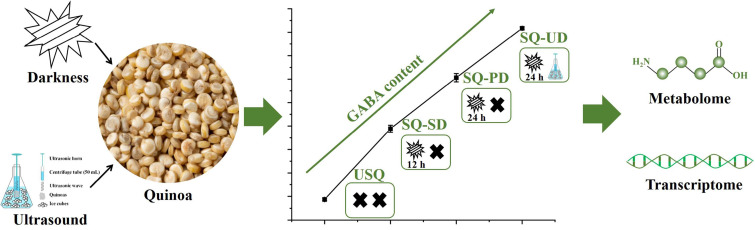
Schematic diagram of the experimental design.

The de-hulled quinoa was sterilized by soaking in 0.1% NaClO for 30 min. Notably, dark and ultrasound stress were identified as potent mechanisms for augmenting GABA levels in quinoa during the germination phase. The disinfected quinoa was soaked in distilled water for 14 h at 24 ± 1 °C. Subsequently, it was incubated in a light-deprived biochemical incubator at the same temperature for 0 h, 12 h, and 24 h to yield sprouted quinoa samples with varying degrees of GABA enrichment under dark stress. These distinct states of unsprouted quinoa, sprouted quinoa under short-term dark stress, and sprouted quinoa under prolonged dark stress were denoted as USQ, SQ-SD, and SQ-PD, respectively. To obtain elevated levels of GABA enrichment in sprouted quinoa under the combined influences of ultrasound and dark stress (SQ-UD), the disinfected quinoa underwent ultrasound exposure using an ultrasonic cell pulverizer from Ningbo Scientz Biotechnology Co., Ltd. (Ningbo, China) at 100 W for 4 min in an ice-water bath, with an ultrasound duration and interval of 3 s each. Following this, the quinoa seeds were soaked in distilled water for 14 h at 24 ± 1 °C and subsequently placed in a light-deprived biochemical incubator at the same temperature for 24 h. The resulting samples were then promptly frozen at −20 °C overnight, subjected to vacuum freeze-drying for 32 h, and subsequently pulverized. The processed samples were stored at −20 °C until further analysis. Based on the preliminary experimental findings, the experimental groups in this study were established according to the observed trend of GABA enrichment. The experimental design is illustrated in Figure 1.

### 2.2. Determination of GABA Content

GABA content was determined via high-performance liquid chromatography [25]. Briefly, the samples were mixed with 0.01 mol/L of citric acid in a ratio of 1:5 (w:w), shaken for 2 h, and centrifuged at 1900× *g* for 15 min. Then, 1 mL of the supernatant, 1 mol/L of NaHCO_3_ (pH = 9), and 0.1% 1-fluoro-2,4-dinitrobenzene acetonitrile solution were mixed thoroughly, incubated in a 60 °C water bath for 90 min, and diluted with 0.01 mol/L of phosphate buffer (pH = 7.2) to 10 mL. GABA content was determined using an Agilent 1260 HPLC equipped with a Universal C18 column (4.6 mm × 250 mm, 5 μm). The mobile phase comprised a mixture of 0.01 mol/L of phosphate buffer (pH = 7.2), ultrapure water, and acetonitrile at a volume ratio of 70:22:8. The flow rate was 1 mL/min, the column temperature was 40 °C, and the GABA peak was detected and quantified at 360 nm.

### 2.3. Widely Targeted Metabolomics Analysis

#### 2.3.1. Metabolite Extraction

The quinoa samples were vacuum freeze-dried (Scientz-100F; Ningbo Sante Biotechnology Co., Ltd., Ningbo, China) and pulverized at 30 Hz for 1.5 min using a mixer mill (MM 400; Retsch, Haan, Germany). Subsequently, 100 mg of lyophilized quinoa powder was dissolved in 1.2 mL of 70% methanol (*v*/*v*), vortexed to mix well, refrigerated at 4 °C for 12 h, and centrifuged at 8500× *g* for 10 min. Finally, the supernatant was used for ultra-high-performance liquid chromatography–tandem mass spectrometry (UPLC-MS/MS) analysis [26].

#### 2.3.2. UPLC and Electrospray Ionization (ESI)–Triple Quadrupole-Linear Ion Trap (Q TRAP)–MS/MS

UPLC-ESI-MS/MS analysis was performed using a Nexera X2 UPLC system (SHIMADZU, Kyoto, Japan) coupled with a 4500 Q TRAP-MS (Applied Biosystems, Foster City, CA, USA). An SB-C18 column (1.8 μm, 2.1 mm × 100 mm; Agilent, Santa Clara, CA, USA) was utilized. Mobile phases A and B were 0.1% aqueous formic acid solution and 0.1% formic acid acetonitrile solution, respectively [27]. The gradient program was as follows: 0–9 min, 95% to 5% A; 9.1–10 min, 5% A; 10.1–11.1 min, 5% to 95% A; 11.1–14.0 min, 95% A. In total, 4 μL of the sample was separated in a 40 °C column at a flow rate of 0.35 mL/min.

A mass spectrometer (Q TRAP AB4500 UPLC/MS/MS System from AB SCIEX Pet. Ltd., Dublin, CA, USA) was used for linear ion trap and triple quadrupole (QQQ) scans and controlled using Analyst 1.6.3 software [28]. The turbine spray was used as the ion source at 550 °C. The ion spray voltages were 5500 V and −4500 V in the positive and negative ion modes, respectively. The pressures of ion source gases I and II and the curtain gas (CUR) were 50, 60, and 25.0 psi, respectively. Multiple reaction monitoring (MRM) experiments were conducted using QQQ scanning with nitrogen gas. Optimized de-clustering potentials and collision energies ensured that each MRM transition was performed. A specific set of MRM transitions was monitored according to the metabolites detected during the assay.

#### 2.3.3. Metabolomics Analysis

Principal component analysis (PCA), hierarchical cluster analysis (HCA), and Pearson’s correlation coefficients (PCC) were performed using R software version 3.5.1 (www.r-project.org). Orthogonal projections to latent structure discriminant analysis (OPLS-DA) results formed the basis for the variable importance in projection (VIP) values [29]. HCA and PCC results were presented as heatmaps with dendrograms, while PCC results were only presented as heatmaps. Differentially expressed metabolites (DEMs) were defined using the criteria VIP ≥ 1 and absolute log_2_fold change (FC) ≥ 1 [30]. Metabolites and metabolic pathways were annotated using the Kyoto Encyclopedia of Genes and Genomes (KEGG) database (http://www.genome.jp/kegg/, accessed on 3 January 2024). The significance of these annotations was determined by the *p*-values of the hypergeometric test.

### 2.4. RNA Sequencing (RNA-Seq)

Fresh quinoa samples from the various treatments were immediately frozen in liquid nitrogen, and total RNA was extracted using pre-chilled TRIzol reagent (Invitrogen, Thermo Fisher Scientific, Waltham, MA, USA), according to the manufacturer’s instructions [31]. A 1% agarose gel was used to monitor RNA degradation and contamination. RNA purity, concentration, and integrity were measured using a NanoPhotometer^®^ spectrophotometer (IMPLEN, Westlake Village, CA, USA), Qubit^®^ RNA Assay Kit (Life Technologies, Carlsbad, CA, USA), and RNA Nano 6000 Assay Kit (Agilent Technologies, Santa Clara, CA, USA) [30], respectively.

RNA samples were prepared using 1 µg of RNA per quinoa sample as input material [31]. A NEBNext^®^ Ultra^TM^ RNA Library Prep Kit (NEB, USA) was used to prepare sequencing libraries. Briefly, 250–300-bp-long cDNA fragments were generated using an AMPure XP System (Beckman Coulter, Beverly, MA, USA). Phusion high-fidelity DNA polymerase, index (X) primers, and universal PCR primers were used for PCR amplification. The PCR products were prepared using an AMPure XP System. An Agilent Bioanalyzer 2100 system was used to assess library quality [31]. To obtain 125 bp/150 bp paired-end reads, a TruSeq PE Cluster Kit v3-cBot HS (Illumina) on the cBot Cluster Generation System and an Illumina HiSeq platform were used for clustering and sequencing, respectively [31]. Fastp v0.19.3 was used to filter the raw data. Data were deemed non-compliant for further analysis when the N content or the number of low-quality bases (Q < 20) in the read base exceeded 10% or 50%, respectively [30].

### 2.5. Differentially Expressed Genes (DEGs) and Gene Function

The resulting clean reads were compared to the reference genome obtained from the HISA T v2.1.0 designated website, using the fragments per kilobase of transcript per million mapped reads (FPKM) method [32]. Gene comparison was performed using Feature Counts v1.6.2, and the FPKM was calculated for each gene based on the gene length. DEG was analyzed using DESeq2 v1.22.1. The DEGs between the treatment groups were identified using the criteria *p*-value < 0.05 and |log_2_FC| > 1 [29]. The hypergeometric distribution tests for KEGG and Gene Ontology (GO) were based on the pathways and GO terms, respectively.

### 2.6. Quantitative Real-Time PCR (qPCR) Validation

Ten DEGs identified through transcriptome sequencing were randomly selected to verify their expression levels via qPCR, using EF1α as an internal control [33]. Table 1 presents the primers used in this experiment. To measure gene expression, quinoa mRNA was extracted and reverse-transcribed using an RNA Easy Fast Plant Tissue Kit and FastKing RT Kit (with gDNase) purchased from Tiangen Biotech Co., Ltd. (Beijing, China). A FastReal qPCR PreMix (SYBR Green) was used on a StepOne Plus Real-Time PCR System (Applied Biosystems, Foster City, CA, USA) [34]. PCR was performed as follows: 95 °C for 2 min, and 40 cycles at 95 °C for 5 s, 55 °C for 10 s, and 72 °C for 15 s. The 2^−ΔΔCt^ method was used to assess the relative expression of each DEG [30].

### 2.7. Determination of Free Amino Acid and Nitrogen Content

The free amino acid content of the samples was analyzed using a Hitachi L 8900 amino acid analyzer (Hitachi, Tokyo, Japan) equipped with a UV detector and a cationic resin chromatography column (200 mm × 4.6 mm) [35]. Briefly, 2 g of each sample was dissolved in 10 mL of water and left to stand for 24 h. Subsequently, 5 mL of 5% sulfosalicylic acid solution was mixed with 5 mL of each sample and centrifuged at 4500× *g* for 10 min. Then, 9 mL of the supernatant was deacidified on a rotary evaporator at 45 °C and dissolved in 1 mL of 1 mol/L sodium citrate buffer. Proline and other amino acids were detected at 440 nm and 570 nm, respectively, with a column temperature of 55 °C [36].

The nitrogen contents were analyzed according to the Chinese standard method GB5009.5-2016.

### 2.8. Statistical Analysis

The results of each test are representative of three replicates. All data in this study are shown as the mean ± standard deviation. We performed one-way analysis of variance (ANOVA) using IBM SPSS Statistics for Windows version 25 (IBM Corp., Armonk, NY, USA) to identify significant differences between sample means. *p* < 0.05 indicated a significant difference, whereas *p* < 0.01 indicated a highly significant difference.

## 3. Results

### 3.1. Enrichment of GABA in Quinoa Under Dark and Ultrasound Stress

**Figure 2:**
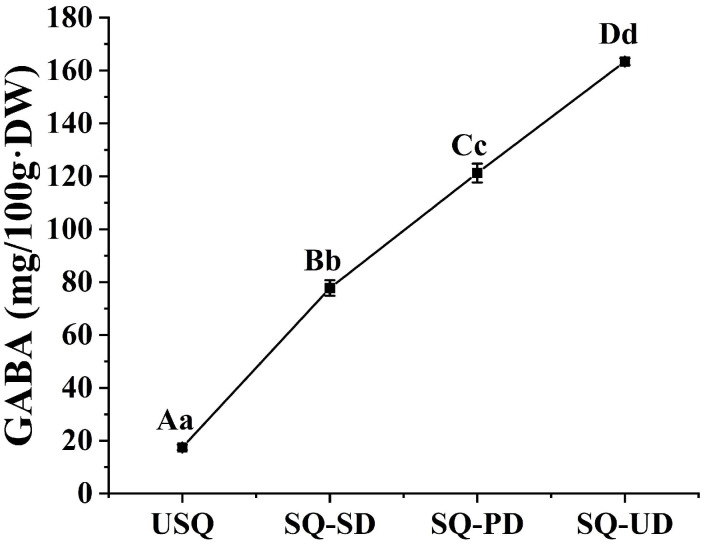
Enrichment of gamma-aminobutyric acid in quinoa under dark and ultrasound stress.

The concentration of GABA in quinoa experienced a significant augmentation under conditions of both dark and ultrasound stress, commencing from an initial GABA level of 17.44 mg/100 g·DW. In comparison to the USQ sample, the GABA content exhibited remarkable increments in the SQ-SD, SQ-PD, and SQ-UD samples by 345.78%, 595.24%, and 836.88%, culminating in concentrations of 77.74 mg/100 g·DW, 121.25 mg/100 g·DW, and 163.39 mg/100 g·DW, respectively (Figure 2).

### 3.2. Metabolomic Analysis of Various Metabolites During GABA Enrichment in Quinoa

**Figure 3:**
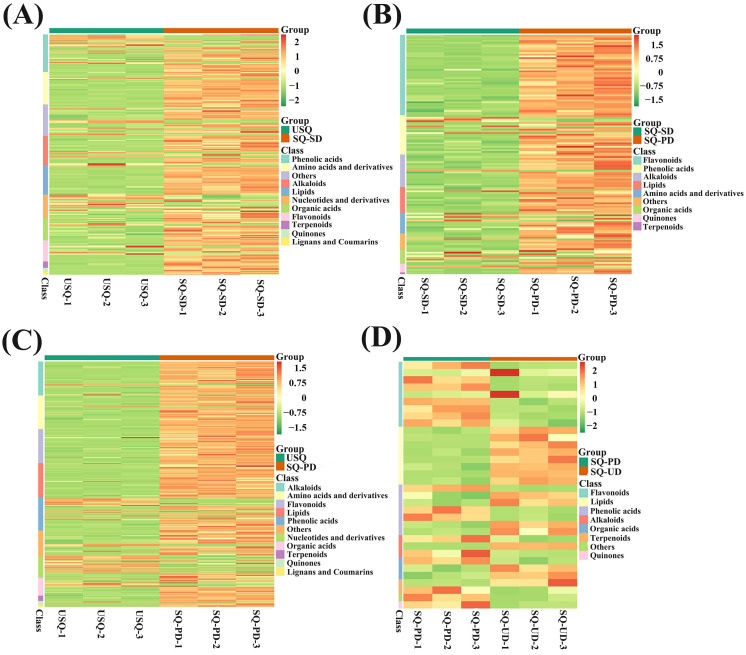
Heatmaps of differential metabolite categories in different quinoa samples: (**A**) USQ vs. SQ-SD, (**B**) SQ-SD vs. SQ-PD, (**C**) USQ vs. SQ-PD, and (**D**) SQ-PD vs. USQ. Colors indicate metabolite accumulation levels from low (green) to high (red).

A comprehensive metabolomics investigation was conducted utilizing UPLC-ESI-MS/MS to scrutinize the metabolic profiles of 4 distinct sets of quinoa samples amidst the process of GABA enrichment under darkness and ultrasound stress, thereby identifying a total of 841 metabolites. Remarkably, significant alterations were observed in 237, 140, 325, and 34 metabolites across the USQ vs. SQ-SD, SQ-SD vs. SQ-PD, USQ vs. SQ-PD, and SQ-PD vs. SQ-UD groups, respectively (Figure S1A). The metabolite compositions in the differently treated quinoa samples were distinctly segregated through PCA, with the biological replicates demonstrating closely clustered patterns, thereby attesting to the exceptional quality and reproducibility of our metabolomics dataset (Figure S1B). Based on the criteria VIP ≥ 1 and absolute log_2_FC ≥ 1, the tally of upregulated and downregulated metabolites in the USQ vs. SQ-SD, SQ-SD vs. SQ-PD, USQ vs. SQ-PD, and SQ-PD vs. SQ-UD groups were 188 and 49, 125 and 15, 274 and 51, and 18 and 16, correspondingly (Figure S2). The heatmap representation (Figure 3) shows that the most recurrently annotated metabolite categories during germination under darkness encompassed flavonoids, alkaloids, lipids, quinones, terpenoids, phenolic acids, amino acids and derivatives, and organic acids. Notably, during germination under ultrasonic stress, the annotated metabolite classes encompassed lipids, flavonoids, quinones, phenolic acids, alkaloids, terpenoids, and organic acids. With regard to DEMs, the analysis revealed upregulation in lipid levels, whereas flavonoids, other compounds, and quinones exhibited downregulation tendencies.

### 3.3. Transcriptomic Analysis of Various Genes During GABA Enrichment in Quinoa

The transcriptome sequencing was conducted utilizing an Illumina HiSeq platform, yielding a total of 535,208,424 clean reads from the 12 quinoa samples. The observed Q30 bases ranged from 93.16% to 94.72%, whereas the GC content percentage fell within the range of 42.99% to 46.33%, indicative of the robust reliability of the transcriptome sequencing outcomes. Noteworthy findings from the PCA of the diverse quinoa samples underscored a high level of reproducibility (Figure S3A). In the first comparison, 9591 genes were upregulated while 11,609 were downregulated, and the second comparison revealed 8733 upregulated and 7954 downregulated genes. Likewise, the third comparison displayed 6600 upregulated and 10,201 downregulated genes, followed by 7096 upregulated and 8535 downregulated genes in the fourth comparison (with absolute log_2_FC ≥ 1 and corrected *p* < 0.05), as delineated in Figures S3B and S4. Additionally, a total of 21,200 DEGs were identified in the paired comparisons between USQ vs. SQ-SD, SQ-SD vs. SQ-PD, USQ vs. SQ-PD, and SQ-PD vs. SQ-UD.

**Figure 4:**
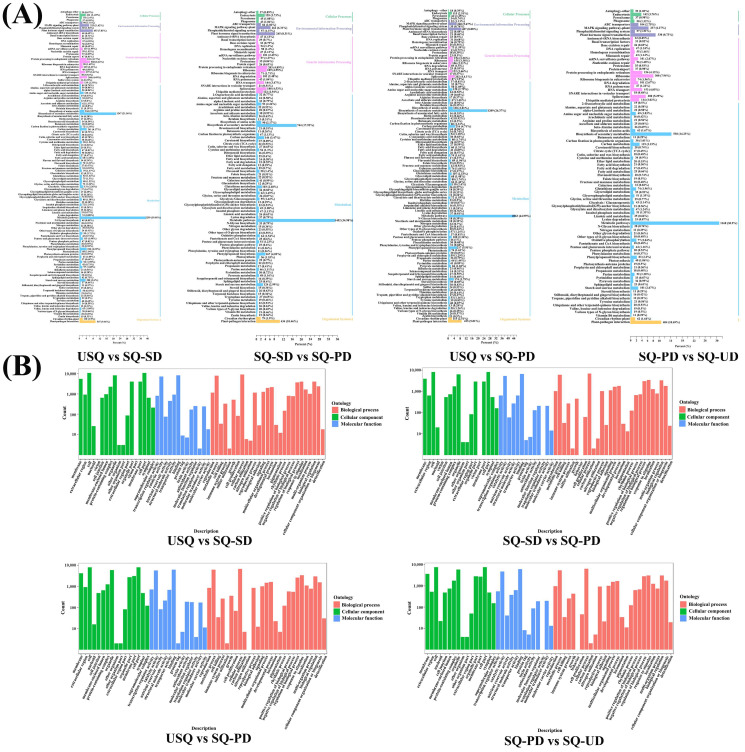
Analysis of differentially expressed genes in different quinoa samples. (**A**) Kyoto Encyclopedia of Genes and Genomes enrichment analysis of all differentially expressed genes. (**B**) Gene Ontology enrichment analysis of all differentially expressed genes.

The KEGG functional annotation of the DEGs across the four comparison groups was scrutinized, revealing pathways predominantly associated with abiotic adversity response, C metabolism, and N metabolism (Figure 4A). Notably, pathways with an annotation percentage exceeding 1% encompassed a spectrum of functions, including plant–pathogen interaction, plant hormone signal transduction, MAPK signaling pathway–plant, endocytosis, phenylpropanoid biosynthesis, glutathione metabolism, and plant circadian rhythm. Moreover, metabolic pathways intertwined with C metabolism encompassed carbon metabolism, glycolysis/gluconeogenesis, carbon fixation in photosynthetic organisms, oxidative phosphorylation, glycerophospholipid metabolism, glyoxylate and dicarboxylate metabolism, pentose and glucuronate interconversion, amino sugar and nucleotide sugar metabolism, starch and sucrose metabolism, and photosynthesis. Similarly, metabolic pathways linked to N metabolism included the ribosome, the spliceosome, protein processing in the endoplasmic reticulum, RNA transport, ubiquitin-mediated proteolysis, ribosome biogenesis in eukaryotes, and amino acid biosynthesis.

Furthermore, GO enrichment analysis of all DEGs underscored their enrichment across diverse molecular functions, biological processes, and cellular components (Figure 4B). The results showed that the significantly enriched GO terms of DEGs in USQ vs. SQ-SD, SQ-SD vs. SQ-PD, and USQ vs. SQ-PD groups were “cellular process”, “metabolic process”, “response to stimulus”, “biological regulation”, and “regulation of biological process”. The significantly enriched GO terms of DEGs in the SQ-PD vs. SQ-UD group were “cellular process”, “metabolic process”, “biological regulation”, “response to stimulus”, and “regulation of biological process”. Notably, the category of biological processes emerged as the most prominent among the four comparison groups, with all five aforementioned items falling under this classification. Regarding molecular function, the DEGs were principally enriched in catalytic activity and binding, while in terms of cellular components, enrichment was primarily observed in the membrane, protein-containing complex, organelle, organelle part, membrane part, and cell part.

### 3.4. Cluster Analysis of Metabolome and Transcriptome During GABA Enrichment in Quinoa

**Figure 5:**
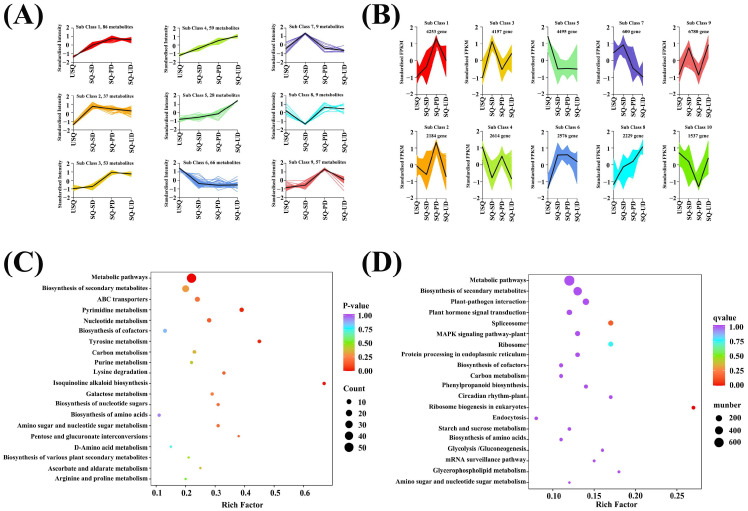
Metabolite and gene expression dynamics in the different quinoa samples. (**A**) Metabolites in quinoa were classified into nine subclasses using the K-means clustering method. (**B**) Genes in the transcriptome of quinoa were classified into 10 subclasses using the K-means clustering method. (**C**) Kyoto Encyclopedia of Genes and Genomes (KEGG) pathway enrichment map for the top 20 most abundantly annotated pathways in subclasses 4 and 6 of the differentially expressed metabolites. (**D**) KEGG pathway enrichment map for the top 20 most abundantly annotated pathways in sub classes 5 and 8 of the differentially expressed genes.

Utilizing a K-means clustering algorithm based on accumulation patterns, all metabolites in quinoa subjected to dark and ultrasonic stress conditions were categorized into nine distinct subclasses (Figure 5A). Upon comparing the accumulation patterns of metabolites across the nine compound subclasses with the trends in GABA enrichment (Figure 2), it was discerned that subclass 4 exhibited a positive correlation, while subclass 6 displayed a negative correlation with GABA enrichment. Subclass 4 encompassed 59 DEMs, including lipids (21), amino acids and derivatives (9), alkaloids (10), phenolic acids (6), organic acids (6), and miscellaneous substances (4). Notably, within this subclass, nine amino acids and their derivatives exhibited a positive correlation with GABA enrichment, including L-valine, L-leucine, *O*-acetylserine, *S*-allyl-L-cysteine, *N*-acetyl-L-leucine, *N*-acetyl-L-glutamine, *N*6-acetyl-L-lysine, 3,4-dihydroxy-L-phenylalanine, and *N*-acetyl-L-tyrosine. Additionally, ten alkaloids and six organic acids were positively associated with GABA enrichment, with alkaloids such as putrescine, triethylamine, cadaverine, indole, isoquinoline, *N*-acetylputrescine, agmatine, 6-deoxyfagomine, dopamine, and *N*-acetyl-5-hydroxytryptamine, and organic acids such as 3-hydroxypropanoic acid, monomethyl succinate, glutaric acid, 2-methylsuccinic acid, D-glucono-1,5-lactone, and 2,6-diaminooimelic acid. Furthermore, four other compounds that demonstrated a positive correlation with GABA enrichment were D-arabinose, rhamnose, D-fructose-1,6-biphosphate, and lactitol. However, subclass 6 comprised 66 DEMs across nucleotides and their derivatives (17), phenolic acids (15), other categories (10), organic acids (7), alkaloids (6), flavonoids (5), amino acids and their derivatives (2), lipids (2), and terpenoids (1). Two amino acids and their derivatives showed an inverse correlation with GABA enrichment: 4-hydroxy-L-glutamic acid and L-methylhistidine. Furthermore, seven organic acids showed an inverse correlation with GABA enrichment: succinic anhydride, 2-hydroxyisobutyric acid, 2-aminoethanesulfinic acid, methylmalonic acid, succinic acid, aminomalonic acid, and 2-aminoethanesulfonic acid. Ten other compounds were inversely correlated with GABA enrichment: dihydroactinidiolide, D-sorbitol, dulcitol, D-galacturonic acid, D-glucuronic acid, *N*-acetyl-D-mannosamine, D-sedoheptulose 7-phosphate, D-sucrose, delta-tocopherol, and raffinose. In Figure S5A, the KEGG enrichment analysis of the DEMs in subclasses 4 and 6 annotated 64 pathways. Within the top 20 most annotated pathways, those linked to C metabolism were notably prevalent, encompassing carbon metabolism, galactose metabolism, biosynthesis of nucleotide sugars, ascorbate and aldarate metabolism, and pentose and glucuronate interconversions. Noteworthy pathways associated with N metabolism included tyrosine metabolism, amino acid biosynthesis, lysine degradation, D-amino acid metabolism, and arginine and proline metabolism. Furthermore, the metabolic pathways of amino sugar and nucleotide sugar were found to be jointly associated with both carbon and nitrogen metabolism (Figure 5C).

Similarly, employing K-means clustering based on accumulation patterns, all DEGs were classified into 10 subclasses (Figure 5B). Among these subclasses, subclasses 5 and 8 exhibited negative and positive correlations with GABA enrichment (Figure 2), respectively. Subsequent analysis of 4495 and 2229 DEGs in subclasses 5 and 8, respectively, using the KEGG enrichment method unveiled 138 pathways annotated in the DEGs (Figure S5B). Notably, pathways associated with C metabolism, such as carbon metabolism, starch and sucrose metabolism, glycolysis/gluconeogenesis, and glycerophospholipid metabolism, were prominent among the top 20 annotated pathways. Additionally, pathways linked to N metabolism, including ribosomes, protein processing in the endoplasmic reticulum, and biosynthesis of amino acids, were identified. Stress-response-related pathways, such as plant–pathogen interaction, plant hormone signal transduction, the MAPK signaling pathway, and circadian rhythm, were also observed. Moreover, amino sugar and nucleotide sugar metabolism were concurrently associated with both carbon and nitrogen metabolism (Figure 5D).

### 3.5. Integration of Metabolomic and Transcriptomic Profiles to Analyze GABA Biosynthesis in Quinoa

Following the K-means analysis, an integrated analytical approach was formulated to explore potential correlations between the metabolomic and transcriptomic variances in quinoa samples with different levels of GABA. By conducting a combined analysis of the KEGG pathways co-annotated between the DEMs and DEGs, distinct associations with GABA enrichment were uncovered. Six shared pathways were identified based on the KEGG functional annotation of the K-means DEMs and DEGs (Figure S5A,B): ko01100 (metabolic pathways), ko01110 (biosynthesis of secondary metabolites), ko01240 (biosynthesis of cofactors), ko01200 (carbon metabolism), ko01230 (biosynthesis of amino acids), and ko00520 (amino sugar and nucleotide sugar metabolism). The K-means analysis unveiled 125 DEMs and 6724 DEGs that were linked to GABA enrichment. The noteworthy KEGG pathways in which GABA was located included ko00250 (alanine, aspartate, and glutamate metabolism), ko00330 (arginine and proline metabolism), ko00410 (beta-alanine metabolism), ko00650 (butanoate metabolism), and ko00760 (nicotinate and nicotinamide metabolism). There were 13 DEMs and 51 annotated DEGs.

**Figure 6:**
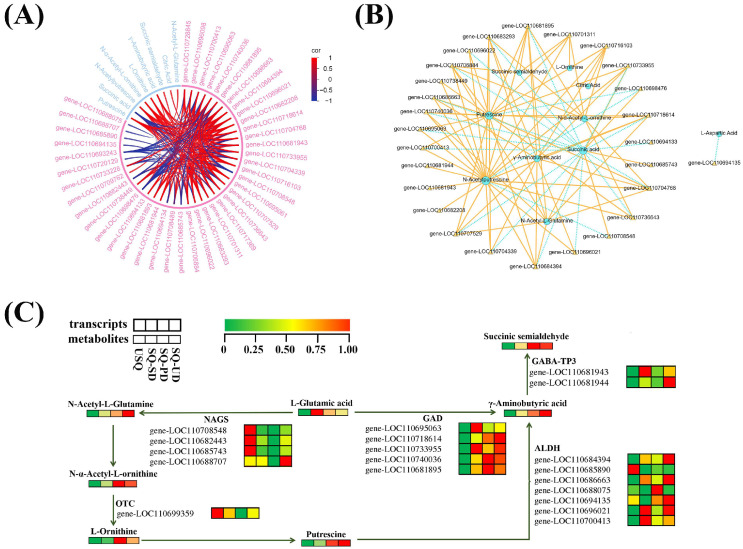
Correlation network and schematic representation of the transcriptomic and metabolic changes in the GABA biosynthetic pathway in quinoa. (**A**) Chord plots of the differentially expressed metabolites (DEMs) and differentially expressed genes (DEGs) associated with GABA enrichment were generated using the R version 3.5.1 software package. (**B**) Network plot of the correlations of key metabolites with DEGs during GABA metabolism in quinoa under dark and ultrasonic stresses. Blue circles and yellow triangles indicate DEMs and DEGs associated with GABA metabolism, respectively. Lines (solid yellow lines for positive correlations and dashed blue lines for negative correlations) indicate correlations between DEMs and DEGs associated with GABA metabolism. (**C**) Schematic representation of the transcriptomic and metabolic changes in the GABA biosynthetic pathway in quinoa. Transcriptomic and metabolic changes in differently treated quinoa samples are shown in heatmaps, where different colors represent the normalized intensities of the corresponding compounds or transcripts. Abbreviations: NAGS, *N*-acetylglutamate synthase; OTC, ornithine carbamoyltransferase; GAD, glutamic acid decarboxylase; ALDH, aldehyde dehydrogenase; GABA-TP3, gamma aminobutyrate transaminase.

Upon scrutinizing the six pathways co-annotated to the K-means DEMs and DEGs, along with the five pathways where GABA is situated (PCC > 0.8, *p* < 0.05), it was discerned that GABA biosynthesis is intricately linked to the equilibrium of carbon and nitrogen. A total of 9 DEMs and 42 DEGs were identified to be closely associated with GABA enrichment (Figure 6A). Notably, the content of putrescine displayed a positive correlation with GABA enrichment, which was further linked to the levels of N-acetyl-L-glutamine, *N*-α-acetyl-L-ornithine, L-ornithine, and *N*-acetylputrescine. Moreover, the estimation of GABA enrichment exhibited positive correlations with six genes, namely, LOC110716103, LOC110704768, LOC110707529, LOC110684394, LOC110686663, and LOC110718614, each contributing to distinct enzymatic processes facilitating GABA production. A comprehensive correlation network analysis unveiled a close association between GABA enrichment and 10 DEMs and 27 DEGs (Figure 6B). GABA enrichment was positively linked to five key genes, including LOC110683293 and LOC110704339 (asparagine synthesis, EC 6.3.5.4), LOC110696021 (aldehyde dehydrogenase, EC 1.2.1.3), and LOC110733955 and LOC110681895 (glutamic acid decarboxylase, EC 4.1.1.15). Furthermore, the chord plots (Figure 6A) and network plots elucidating the correlations of pivotal metabolites with DEGs (Figure 6B) underscored a robust association between GABA enrichment and 9 DEMs, alongside 27 DEGs.

The depiction in Figure 6C outlines the GABA biosynthesis pathways in quinoa under dark and ultrasonic stress conditions. The results indicate that GABA is directly synthesized from L-glutamic acid and putrescine through two pathways. Initially, L-glutamic acid is converted into GABA by the action of GAD (EC 4.1.1.15). Alternatively, L-glutamic acid may be transformed into putrescine through the enzymatic activities of *N*-acetylglutamate synthase (NAGS, EC 2.3.1.1) and ornithine carbamoyltransferase (OTC, EC 2.6.1.13), which are subsequently converted into GABA by aldehyde dehydrogenase (ALDH, EC 1.2.1.3). GABA is then transformed into succinic semialdehyde under the catalysis of gamma aminobutyrate transaminase (GABA-T3, EC 2.6.1.96). Furthermore, L-aspartic acid can be converted into L-asparagine under the catalytic action of asparagine synthetase (AS, EC 6.3.5.4). L-asparagine can then be transformed into oxaloacetate, entering the citric acid cycle, where it is gradually converted into citric acid and 2-oxoglutarate. The latter is further processed by glutamate dehydrogenase (GDH, EC 1.4.1.3) and glutamate synthase (GOGAT, EC 1.4.1.14) to produce L-glutamic acid. This biological process, represented in the ko00250 pathway, provides L-glutamic acid as a precursor for the synthesis of GABA.

### 3.6. Expression Patterns and Validation of the GABA Biosynthetic Pathway Genes

**Figure 7:**
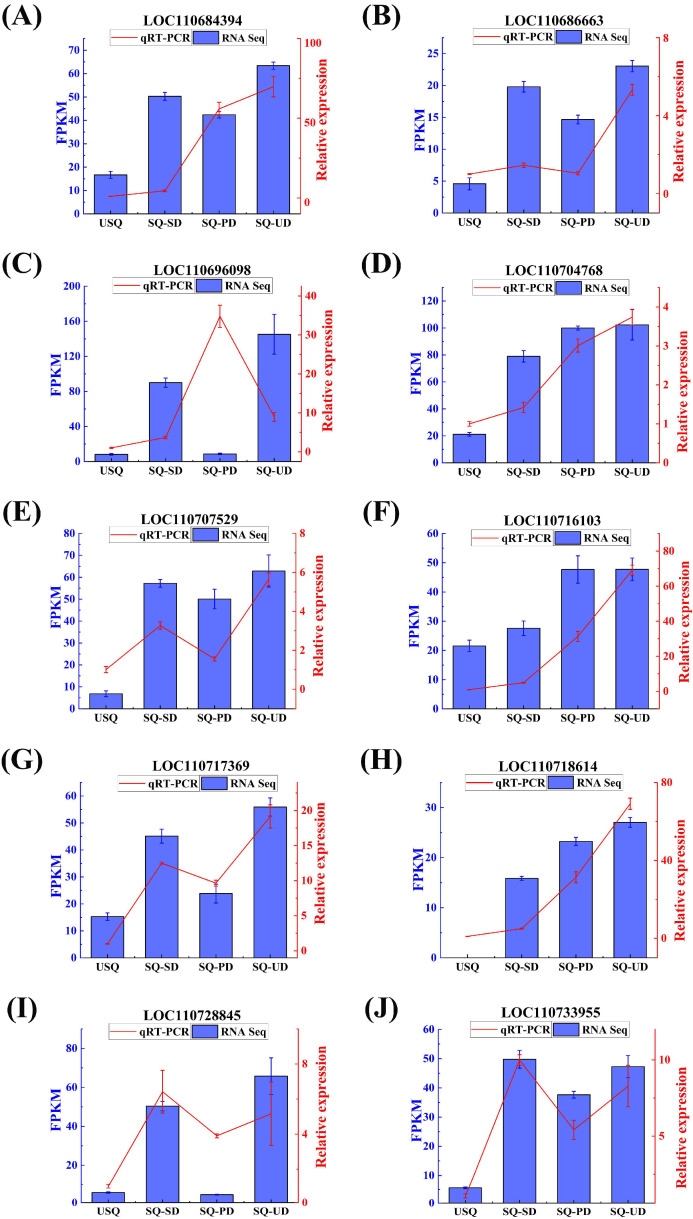
Expression trends of 10 differentially expressed genes validated using qPCR. The blue bar chart corresponds to the blue y-axis, representing the expression levels of individual genes in transcriptomics. The red line graph aligns with the red y-axis, depicting the expression levels of individual genes, as determined by qPCR. (**A**) LOC110684394. (**B**) LOC110686663. (**C**) LOC110696098. (**D**) LOC110704768. (**E**) LOC110707529. (**F**) LOC110716103. (**G**) LOC110717369. (**H**) LOC110718614. (**I**) LOC110728845. (**J**) LOC110733955.

The expression trends of 10 DEGs (FPKM > 10) associated with GABA synthesis were verified via qPCR. Figure 7 shows that DEGs were consistent with the expression trend observed in RNA-Seq, indicating that the RNA-Seq data are reliable for analysis.

### 3.7. Amino Acid Profile Related to GABA Enrichment in Quinoa

In the metabolite summaries derived from the K-means analyses of categories 4 and 6, a notable association emerged between amino acid metabolism, encompassing valine, leucine, serine, cysteine, glutamate, and their derivatives, and the process of GABA enrichment. Pathways, such as arginine biosynthesis (ko00220), alanine, aspartate, and glutamate metabolism (ko00250), and arginine and proline metabolism (ko00330), were identified as closely intertwined with GABA metabolism (Figure 6). The effects of darkness and ultrasound stress on the levels of free amino acids in quinoa are shown in Table 2. The comparative analysis between SQ-PD and USQ shows the impact of darkness, and the comparative analysis between SQ-UD and SQ-PD reveals the impact of ultrasound. The results revealed that dark stress had no substantial impact on the methionine levels; however, it induced a pronounced increase in the concentrations of aspartate, threonine, serine, glutamate, glycine, alanine, cysteine, valine, isoleucine, leucine, tyrosine, phenylalanine, histidine, lysine, arginine, and proline. On the other hand, ultrasonic stress caused a significant reduction in the levels of glutamate and arginine, while notably enhancing the concentrations of aspartate, threonine, serine, glycine, alanine, cysteine, valine, methionine, isoleucine, leucine, tyrosine, phenylalanine, histidine, lysine, and proline. Both dark and ultrasonic stresses led to a significant increase in the overall free amino acid content, while having no significant effect on the nitrogen content of quinoa samples (Figure S6). In conclusion, darkness and ultrasonic stress were advantageous for the synthesis of amino acids.

### 3.8. Changes in Phytochemical Content During GABA Enrichment

**Figure 8:**
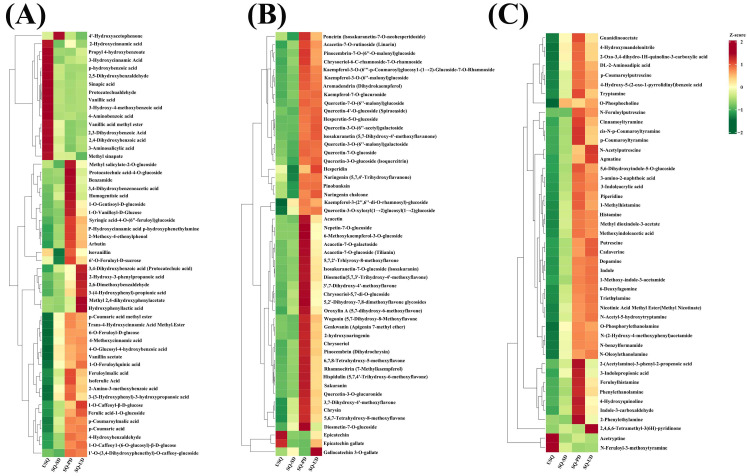
Heatmap analysis of the differential phenolic acids (**A**), flavonoids (**B**), and alkaloids (**C**).

Germination processes under dark and ultrasound stress conditions have the potential to amplify the enrichment of phytochemical compounds. In this investigation, notable fluctuations in the levels of phenolic acids, flavonoids, and alkaloids were observed in quinoa exposed to dark and ultrasonic stresses, as illustrated in Figure 8A–C, respectively. Specifically, a total of 53 phenolic acids, 51 flavonoids, and 45 alkaloids with VIP ≥ 1.0 were identified. Among these compounds, six phenolic acid varieties (including 3,4-dihydroxybenzoic acid, 2-hydroxy-3-phenylpropanoic acid, 2,6-dimethoxybenzaldehyde, 3-[4-hydroxyphenyl]-propionic acid, methyl 2,4-dihydroxyphenylacetate, and hydroxyphenyllactic acid), one flavonoid (gallocatechin 3-*O*-gallate), and one alkaloid (2,4,6,6-tetramethyl-3(6H)-pyridinone) exhibited significant enrichment after ultrasonic stress, possibly attributable to the reinforcement of the stress response in quinoa. The investigation delved into the top 20 metabolites within each of these 3 categories of compounds, focusing on the differential phenolic acids, flavonoids, and alkaloids that exhibited distinct expression levels at SQ-UD in comparison to USQ. Among these, six phenolic acids (such as 2-methoxy-4-vinylphenol, ferulic acid-1-*O*-glucoside, arbutin, protocatechuic acid, vanillin acetate, and 1-*O*-feruloylquinic acid), seven flavonoids (including nepetin-7-*O*-glucoside, 6-methoxykaempferol-3-*O*-glucoside, chrysin, chrysoeriol-5,7-di-*O*-glucoside, 5,6,7-tetrahydroxy-8-methoxyflavone, kaempferol-3-(2′,6′-di-*O*-rhamnosyl)-glucoside, and 5,2′-dihydroxy-7,8-dimethoxyflavone glycosides), and nine alkaloids (such as 2,4,6,6-tetramethyl-3(6H)-pyridinone, 5,6-dihydroxyindole-5-*O*-glucoside, *N*-acetylputrescine, methyl dioxindole-3-acetate, agmatine, triethylamine, 2-oxo-3,4-dihydro-1H-quinoline-3-carboxylic acid, methyl nicotinate, and *N*-feruloylputrescine) were notably absent at USQ and exhibited enrichment after dark germination and ultrasonic stress. The enrichment values for the remaining 14 phenolic acids, 13 flavonoids, and 11 saponins varied from 3.26 to 28.67, 5.73 to 35.47, and 4.58 to 30.77, respectively.

## 4. Discussion

GABA stands as a vital nonprotein amino acid abundantly present in various food sources. Renowned for its diverse array of health benefits encompassing neuroprotection, alleviation of depressive symptoms and insomnia, and potential advantages for individuals grappling with diabetes and hypertension owing to its anti-inflammatory properties [16], GABA emerges as a pivotal dietary component. Notably, GABA enrichment is a characteristic response observed in plants under abiotic stresses [11], a phenomenon mirrored in our experimental findings showcasing an 836.88% increase in GABA content in quinoa subjected to dark and ultrasound stresses (Figure 2). Nonetheless, the precise impact of dark and ultrasonic stresses on the GABA metabolic pathway in quinoa during germination remains shrouded in ambiguity. Thus, leveraging metabolomic and transcriptomic synergies, we endeavored to unravel the intricate molecular mechanisms underpinning GABA enrichment in quinoa exposed to dark and ultrasonic stresses. This elucidation holds paramount significance for the advancement of whole-grain functional foods teeming with GABA. Concurrently, our exploration delved into unraveling the effects of dark stress and ultrasound stress on the phytochemical composition of quinoa during the germination process.

The heatmap analysis illustrated the accumulation of flavonoids, lipids, terpenoids, and quinones in quinoa during germination under dark stress (Figure 3). Conversely, distinct patterns emerged during germination under ultrasonic stress, with differentially expressed lipids exhibiting an upregulation trend, whereas differentially expressed flavonoids, among others, and quinones showcased a downregulation pattern. Flavonoids, recognized as pivotal secondary metabolites, are instrumental in plant resilience against abiotic stressors [37]. The present study unveiled substantial variations in transcript levels across diverse quinoa samples. Notably, the KEGG functional annotation outcomes highlighted that the pathways enriched in differential gene expression predominantly revolved around responses to abiotic challenges and the maintenance of metabolic equilibrium in carbon and nitrogen (Figure 4A). Furthermore, GO enrichment analyses unveiled that the DEGs annotated to biological processes, particularly those associated with responses to stimuli, exhibited notable enrichment levels among the four treatment groups (Figure 4B).

The K-means clustering analysis delineated the classification of all DEMs and DEGs into nine and ten subclasses, respectively, predicated on their distinct accumulation patterns, as illustrated in Figure 5A,B. Leveraging an integration of data derived from both the metabolome and transcriptome, we aimed to deepen our understanding of the metabolic intricacies, pivotal catalytic enzymes, and regulatory elements governing GABA in quinoa species subjected to dark and ultrasonic stresses. By intertwining this information with the metabolic pathways housing GABA, we unearthed that the enrichment of GABA in quinoa under the influence of dark and ultrasonic stresses was intimately linked with the delicate balance of carbon and nitrogen metabolism, hormone signaling, and stress response. Previous studies have indicated that GABA assumes a crucial role in bridging amino acid metabolism with the TCA cycle to finely regulate the fluxes of carbon and nitrogen metabolism in plants [18]. This finding aligns with our experimental results. Previous research on wheat leaves has revealed that under salt stress conditions, the TCA cycle encounters inhibition. Our findings align with the notion that the GABA shunt pathway serves as a supplementary carbon source for fueling the TCA cycle [38]. Our research findings indicated that the biosynthesis of GABA primarily involves two pathways: L-glutamic acid is converted into GABA under the influence of GAD; alternatively, L-glutamic acid can be converted into putrescine through the enzymatic activities of NAGS and OTC, which is subsequently transformed into GABA by ALDH. The first pathway is the GABA shunt pathway reported by previous researchers, and GAD is the rate-limiting enzyme in this biological process [39,40]. Our previous experimental data also indicated a positive correlation between GABA activity and GAD [25]. Additionally, the conversion of glutamate to putrescine, eventually culminating in GABA production under the catalytic influence of ALDH, underscored the significance of the polyamine pathway in driving GABA enrichment in quinoa. Notably, compared to normal embryo rice grains, the heightened GABA content observed in giant embryo rice grains is primarily attributed to the activation of the polyamine pathway and the downregulation of GABA catabolism [39], emphasizing the critical role played by the polyamine pathway in modulating GABA levels. Similarly, under hypoxic stress conditions, a notable proportion of GABA in tea plants is synthesized through the polyamine degradation pathway [41], where diamine oxidase (DAO) facilitates the conversion of putrescine to GABA [18,42]. Our study further unveiled the novel finding that ALDH catalyzes the conversion of putrescine to generate GABA in quinoa, shedding light on a less-explored facet of GABA biosynthesis pathways.

In the integrated metabolomic and transcriptomic analyses, a set of six genes, namely, LOC110716103, LOC110704768, LOC110707529, LOC110684394, LOC110686663, and LOC110718614, emerged as positively correlated with GABA enrichment, as visually depicted in the chord plot (Figure 6A) and the comprehensive correlation network plot (Figure 6B). Among these genes, LOC110707529, LOC110686663 (ALDH, EC 1.2.1.3), and LOC110718614 (GAD, EC 4.1.1.15) were directly involved in catalyzing GABA production from putrescine and glutamic acid, respectively (Figure 6C), which may serve as rate-limiting factors in the GABA production pathway in quinoa under the influence of dark and ultrasonic stresses.

**Figure 9:**
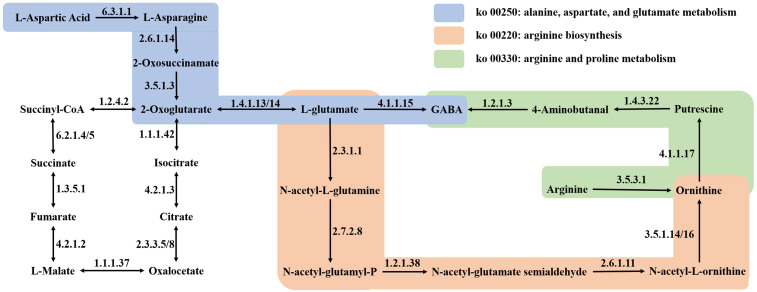
Enrichment mechanism of GABA in quinoa under dark and ultrasound stresses.

The biosynthesis of GABA is intricately intertwined with both the synthetic and catabolic pathways of amino acids. Similarly, investigations into the accumulation of GABA during the germination of coix revealed a profound connection between GABA accumulation and amino acid metabolism [18]. Notably, glutamate could undergo sequential conversions to ornithine and putrescine, eventually leading to GABA generation within the context of arginine biosynthesis (ko00220) and alanine, aspartate, and glutamate metabolism (ko00250). This metabolic cascade potentially elucidated the observed decline in glutamate levels noted at SQ-UD. Preceding GABA synthesis, arginine could be metabolized to ornithine and putrescine through the arginine and proline metabolism pathway (ko00330). This process provides the necessary precursor, putrescine, for the accumulation of GABA in quinoa, while also explaining the reduction in arginine content under ultrasonic stress conditions. Similarly, plants also promote the aforementioned process to accumulate GABA in response to cold stress [18]. Although the metabolomic outcomes may not distinctly reflect these dynamics because of their lower significance levels, it is essential to recognize the intricate interplay within these metabolic pathways. In addition, aspartic acid is linked to the GABA pathway through the TCA cycle in the alanine, aspar-tate, and glutamate metabolism (ko00250) pathway. Specifically, L-aspartic acid can be converted into L-asparagine under the catalytic action of AS. L-asparagine then transforms into oxaloacetate, entering the citric acid cycle, where it is gradually converted into citric acid and 2-oxoglutarate. The latter is further processed by GDH and GOGAT to produce L-glutamate, which serves as a precursor for the synthesis of GABA (Figure 9). This is due to the fact that aspartic acid, as a glucogenic amino acid, is capable of contributing to the metabolic processes involving glutamic acid [43]. Proline accumulation has been linked to conferring tolerance against abiotic stressors [44,45], underscoring its pivotal role in stress response mechanisms. Noteworthy findings on alanine highlight its role in promoting cellular energy metabolism in germinated rice [13], showcasing expression patterns akin to GABA under ultrasonic treatment. The pathway of alanine, aspartate, and glutamate metabolism (ko00250) suggests that alanine can be metabolized to pyruvate, thus fostering energy metabolism within the cellular milieu. Overall, darkness and ultrasonic stress fostered amino acid synthesis without markedly influencing nitrogen content in quinoa samples. Prior experimental studies suggested this phenomenon might stem from the fragmentation of protein peptide chains and the mutual conversion of amino acids [25].

Notably, germination under dark and ultrasound stress conditions resulted in different levels of enrichment in phenolic acids, flavonoids, and alkaloid metabolites (Figure 8). Polyphenols and flavonoids, renowned for their antioxidative properties, have been documented to mitigate the adverse impacts of abiotic stress on plants [46,47]. Our findings indicated that ultrasound stress elicited an increase in the content of 2-hydroxy-3-phenylpropionic acid. Intriguingly, similar observations of enhanced 2-hydroxy-3-phenylpropionic acid content have been reported in *Rhododendron chrysanthum* Pall. under UV-B stress [48]. Furthermore, germination under dark and ultrasound stress conditions manifested a notable abundance of arbutin in quinoa. Arbutin, recognized for its antioxidant properties, has been linked to bolstering drought resistance in safflower and salt tolerance in rice [49,50], underpinning its potential benefits in stress mitigation. Additionally, following germination and exposure to UV-B radiation, the content of 3,4-dihydroxybenzoic acid in riceberry rice and barley exhibited an increase, serving as a protective shield against detrimental environmental stressors [51,52]. This aligns with our research findings, further substantiating the role of specific phytochemical compounds in safeguarding plants from adverse environmental effects.

## 5. Conclusions

These findings confirmed the potential of ultrasound pretreatment for dark germination in enhancing GABA enrichment in quinoa and elucidated its mechanisms of action at both the gene and metabolite levels. We proved that quinoa exhibits dual pathways for GABA synthesis: one involving the conversion of glutamate to GABA catalyzed by GAD, and the other entailing the transformation of putrescine to GABA catalyzed by ALDH. Notably, LOC110707529, LOC110686663, and LOC110718614 were identified as potential rate-limiting genes governing GABA enrichment in quinoa under dark and ultrasonic stresses. The enrichment of GABA demonstrated a close association with amino acid biosynthesis and degradation processes, with heightened levels of proline and alanine observed under these stress conditions, contrasted by decreased levels of glutamate and arginine. Moreover, the study revealed an enrichment of phenolic acids, flavonoids, and alkaloid metabolites in the quinoa samples. These findings offer valuable insights into genetic resources for potential metabolic engineering endeavors aimed at modulating GABA levels in quinoa, alongside providing theoretical underpinnings for the advancement of quinoa-based processing techniques. Future research avenues could potentially explore the regulatory mechanisms governing the key enzymes and genes identified in the GABA biosynthetic pathway, with scope for functional validation experiments to elucidate the roles of specific genes and metabolites in GABA enrichment. Overall, the combination of dark and ultrasound stress treatments is a promising approach in the agricultural food industry to increase the accumulation of beneficial and healthy components in sprouted grains.

## Figures and Tables

**Table 1:** Primer sequences designed for quantitative real-time PCR.

Gene ID	Forward Primer (5′ to 3′)	Reverse Primer (5′ to 3′)
LOC110718614	CAGCAAGTGTCTGTTTTTCTAATCG	CGCGTTCTCTTGGCAGTTTT
LOC110733955	ATACGGCTTGGCTTTGAGGT	GAGCCATGGACCACTATTTTGC
LOC110684394	AGGCATCTTATGACAAGGTAAAAGC	CCTGTGCAGAAGCGTCAAAG
LOC110686663	TGTGAGTGAAAGTGTTGTCCCA	ACGACCAAGGAGGTTCCAC
LOC110681943	ACATCAGTGCAGCAAACAATAGTC	TAGGGGTGCTAACACAAGCAG
LOC110704768	CCAAAAGTCGGGATGCTTGC	GCAGTTGCTCATCTTTCTGTCC
LOC110707529	GTGTTGTCATCCTTCCCGACA	AGTGGAGGATTTCATACCTGAACC
LOC110728845	AATGGGAGAGGGGGACTAGAAT	CAAGGACTGCCCTCGTCTAC
LOC110696098	ACTCCAACCTCACTTGCCATT	TTTCCCTGCTGAGTGCCTG
LOC110717369	TTTCCCGTGTTGTCCTCATCC	CAACACTTGGACCACCTGGA
LOC110716103	GAGATGTGGGGTGCCACTTT	TTGCTTTGTGTAAGTCAACGAGAC
EF1α	GTACGCATGGGTGCTTGACAAACTC	ATCAGCCTGGGAGGTACCAGTAAT

**Table 2:** Differences in the levels of free amino acids in quinoa samples under dark and ultrasonic stresses ^1^.

Amino Acids (mg/g)	USQ	SQ-SD	SQ-PD	SQ-UD
Aspartate	0.242 ± 0.020 ^Aa^	0.329 ± 0.048 ^Bb^	0.295 ± 0.017 ^ABb^	0.418 ± 0.014 ^Cc^
Threonine	0.173 ± 0.016 ^Aa^	0.189 ± 0.010 ^AaB^	0.222 ± 0.011 ^Bb^	0.322 ± 0.012 ^Cc^
Serine	0.321 ± 0.018 ^Aa^	0.474 ± 0.018 ^Bb^	0.415 ± 0.008 ^Cc^	0.371 ± 0.014 ^Dd^
Glutamate	0.555 ± 0.024 ^Aa^	0.839 ± 0.044 ^Bb^	0.721 ± 0.017 ^Cc^	0.627 ± 0.011 ^Ad^
Glycine	0.177 ± 0.012 ^Aa^	0.097 ± 0.008 ^Bb^	0.103 ± 0.014 ^Bb^	0.176 ± 0.012 ^Aa^
Alanine	0.245 ± 0.023 ^Aa^	0.263 ± 0.013 ^Aa^	0.367 ± 0.018 ^Bb^	0.467 ± 0.010 ^Cc^
Cysteine	0.149 ± 0.008 ^Aa^	0.130 ± 0.010 ^Ab^	0.184 ± 0.010 ^Bc^	0.263 ± 0.008 ^Cd^
Valine	0.313 ± 0.011 ^Aa^	0.327 ± 0.024 ^Aa^	0.394 ± 0.004 ^Bb^	0.548 ± 0.013 ^Cc^
Methionine	0.195 ± 0.011 ^Aa^	0.190 ± 0.012 ^Aa^	0.200 ± 0.009 ^Aa^	0.405 ± 0.006 ^Bb^
Isoleucine	0.242 ± 0.015 ^Aa^	0.256 ± 0.017 ^Aa^	0.324 ± 0.018 ^Bb^	0.439 ± 0.011 ^Cc^
Leucine	0.305 ± 0.007 ^Aa^	0.323 ± 0.014 ^Aa^	0.427 ± 0.009 ^Bb^	0.613 ± 0.009 ^Cc^
Tyrosine	0.294 ± 0.007 ^Aa^	0.306 ± 0.006 ^Aa^	0.377 ± 0.008 ^Bb^	0.601 ± 0.015 ^Cc^
Phenylalanine	0.239 ± 0.011 ^Aa^	0.232 ± 0.016 ^Aa^	0.298 ± 0.012 ^Bb^	0.522 ± 0.011 ^Cc^
Histidine	0.189 ± 0.010 ^Aa^	0.287 ± 0.012 ^Bb^	0.329 ± 0.008 ^Cc^	0.367 ± 0.010 ^Dd^
Lysine	0.281 ± 0.015 ^Aa^	0.283 ± 0.012 ^Aa^	0.420 ± 0.012 ^Bb^	0.697 ± 0.011 ^Cc^
Arginine	0.604 ± 0.014 ^Aa^	0.729 ± 0.015 ^Bb^	0.807 ± 0.013 ^Cc^	0.601 ± 0.018 ^Aa^
Proline	0.309 ± 0.012 ^Aa^	0.298 ± 0.011 ^Aa^	0.395 ± 0.004 ^Bb^	0.524 ± 0.010 ^Cc^
Total amino acids	4.832 ± 0.010 ^Aa^	5.553 ± 0.021 ^Bb^	6.278 ± 0.046 ^Cc^	7.957 ± 0.013 ^Dd^

^1^ In the same row, different lowercase and capital letters indicate significant and extremely significant differences at *p* < 0.05 and *p* < 0.01, respectively. The data reported in this chart are expressed as the mean ± standard deviation (*n* = 3).

## Data Availability

The original contributions presented in the study are included in the article/Supplementary Material, further inquiries can be directed to the corresponding authors.

## Supplementary Materials

The following supporting information can be downloaded at: https://www.mdpi.com/article/10.3390/foods14071186/s1. Figure S1: Venn analysis (A) and principal component analysis (B) of the metabolites in the different quinoa samples. Figure S2: Volcano maps of the metabolites in the different quinoa samples. (A) USQ vs. SQ-SD. (B) SQ-SD vs. SQ-PD. (C) USQ vs. SQ-PD. (D) SQ-PD vs. USQ. Red, green and gray points represent the enriched, reduced, and not significantly different metabolites, respectively. Figure S3: Principal component analysis (A) and Venn diagram (B) of the transcriptome in the different quinoa samples. Figure S4: Volcano maps of the transcriptome in the different quinoa samples. (A) USQ vs. SQ-SD. (B) SQ-SD vs. SQ-PD. (C) USQ vs. SQ-PD. (D) SQ-PD vs. USQ. Red, green, and blue points in the volcano maps represent enriched, reduced, and not significantly different differentially expressed genes, respectively. Figure S5: Metabolite and gene expression dynamics in the different quinoa samples. (A) Kyoto Encyclopedia of Genes and Genomes (KEGG) classification plot of the differentially expressed metabolites in sub classes 4 and 6. (B) KEGG classification plot of the differentially expressed genes in sub classes 5 and 8. Figure S6: Differences in the levels of nitrogen content in the different quinoa samples. Different lowercase and capital letters indicate significant and extremely significant differences at *p* < 0.05 and *p* < 0.01, respectively.
