# Supplementary material for: Integrated Metabolomic and Transcriptomic Analysis Reveals the Molecular Regulatory Mechanism of Gamma-Aminobutyric Acid Accumulation in White Quinoa (Chenopodium quinoa Willd.) in Response to Dark and Ultrasound Stress
Source: Foods. 2025 Mar 28;14(7):1186. doi: 10.3390/foods14071186 (PMC11988790; doi:10.3390/foods14071186)
Supplement: Supplementary file 1 [file foods-14-01186-s001.zip › revised-foods-3528854-supporting information.pdf]

Supporting Information

Integrated metabolomic and transcriptomic analysis reveals the molecular regulatory mechanism of gamma-aminobutyric acid accumulation in white quinoa (*Chenopodium quinoa* Willd.) in response to dark and ultrasound stress

Mengying Wu [1], Qian Zhou [1], Yasai Sun [1], Liangfu Zhou [1], Dongyao Li [1], Ting Ren [1], Yu Zheng [1], Wen Zhao [1,*] and Jie Wang [1,*]

[1] College of Food Science and Technology, Agricultural University of Hebei, Baoding 071001, China

[*] Correspondence

Wen Zhao – Tel/Fax: +86 13582820221; Email: zhaowen@hebau.edu.cn

Jie Wang – Tel/Fax: +86 13131262819; Email: wangjie@hebau.edu.cn

E-mail addresses: wumy9902@163.com (Mengying Wu), zhouqian@hebau.edu.cn (Qian Zhou), sunyasai@hebau.edu.cn (Yasai Sun), zhoulf202201@163.com (Liangfu Zhou), lidongyao@hebau.edu.cn (Dongyao Li), rt1134771972@163.com (Ting Ren), zhengyu9818@163.com (Yu Zheng), zhaowen@hebau.edu.cn (Wen Zhao), wangjie@hebau.edu.cn (Jie Wang).

Figure captions

Figure S1. Venn analysis (A) and principal component analysis (B) of the metabolites in the different quinoa samples.

Figure S2. Volcano maps of the metabolites in the different quinoa samples. (A) USQ vs SQ-SD. (B) SQ-SD vs SQ-PD. (C) USQ vs SQ-PD. (D) SQ-PD vs USQ. Red, green and gray points represent the enriched, reduced, and not significantly different metabolites, respectively.

Figure S3. Principal component analysis (A) and Venn diagram (B) of the transcriptome in the different quinoa samples.

Figure S4. Volcano maps of the transcriptome in the different quinoa samples. (A) USQ vs SQ-SD. (B) SQ-SD vs SQ-PD. (C) USQ vs SQ-PD. (D) SQ-PD vs USQ. Red, green, and blue points in the volcano maps represent enriched, reduced, and not significantly different differentially expressed genes, respectively.

Figure S5. Metabolite and gene expression dynamics in the different quinoa samples. (A) Kyoto Encyclopedia of Genes and Genomes (KEGG) classification plot of the differentially expressed metabolites in sub classes 4 and 6. (B) KEGG classification plot of the differentially expressed genes in sub classes 5 and 8.

Figure S6. Differences in the levels of nitrogen content in the different quinoa samples. Different lowercase and capital letters indicate significant and extremely significant differences at $p < 0.05$ and $p < 0.01$, respectively.

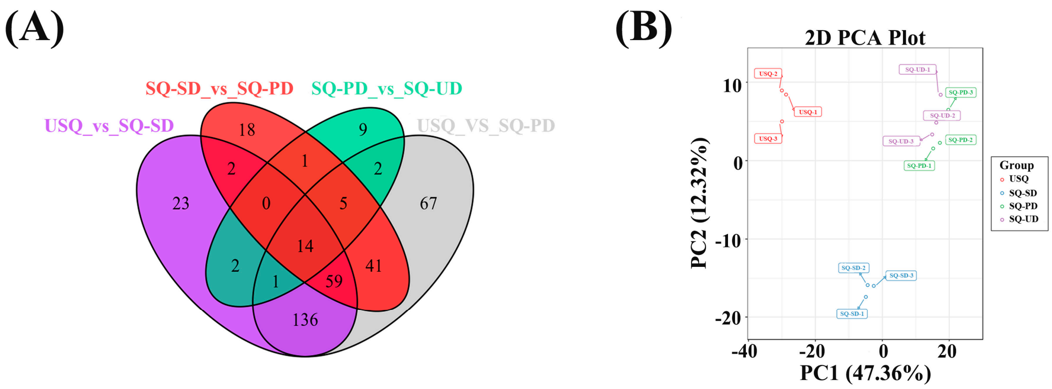

Figure S1. Venn analysis (A) and principal component analysis (B) of the metabolites in the different quinoa samples.

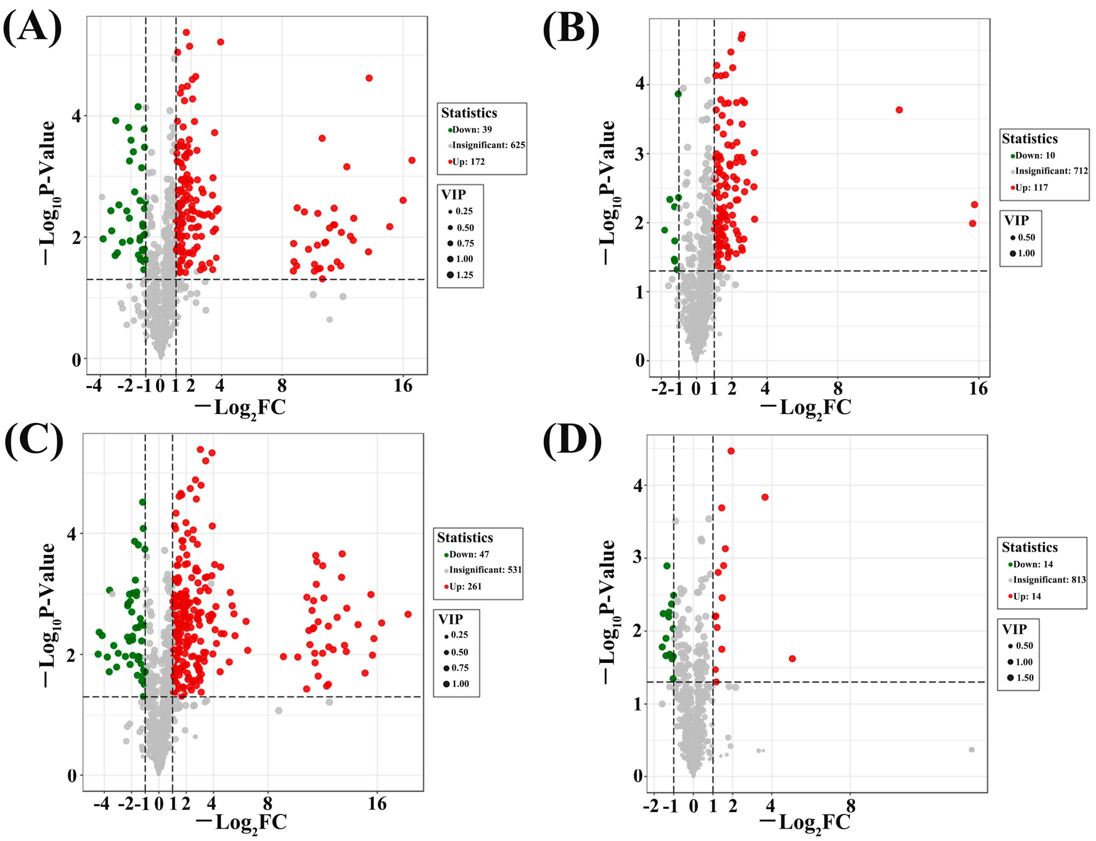

Figure S2. Volcano maps of the metabolites in the different quinoa samples. (A) USQ vs SQ-SD. (B) SQ-SD vs SQ-PD. (C) USQ vs SQ-PD. (D) SQ-PD vs USQ. Red, green and gray points represent the enriched, reduced, and not significantly different metabolites, respectively.

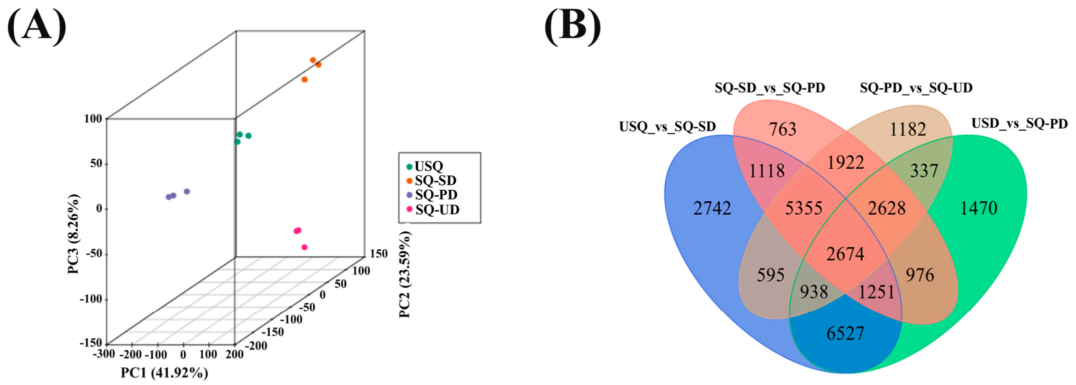

Figure S3. Principal component analysis (A) and Venn diagram (B) of the transcriptome in the different quinoa samples.

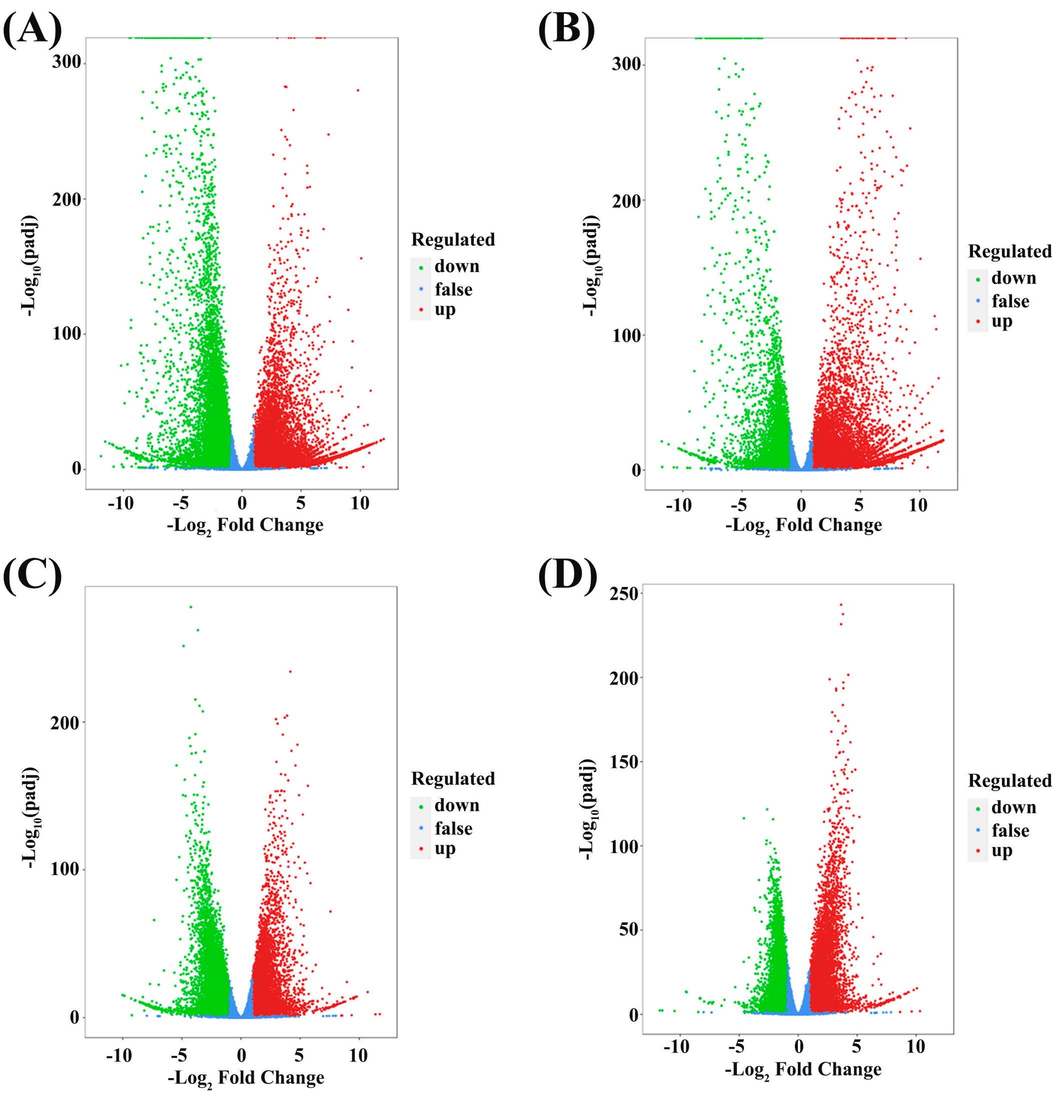

Figure S4. Volcano maps of the transcriptome in the different quinoa samples. (A) USQ vs SQ-SD. (B) SQ-SD vs SQ-PD. (C) USQ vs SQ-PD. (D) SQ-PD vs USQ. Red, green, and blue points in the volcano maps represent enriched, reduced, and not significantly different differentially expressed genes, respectively.

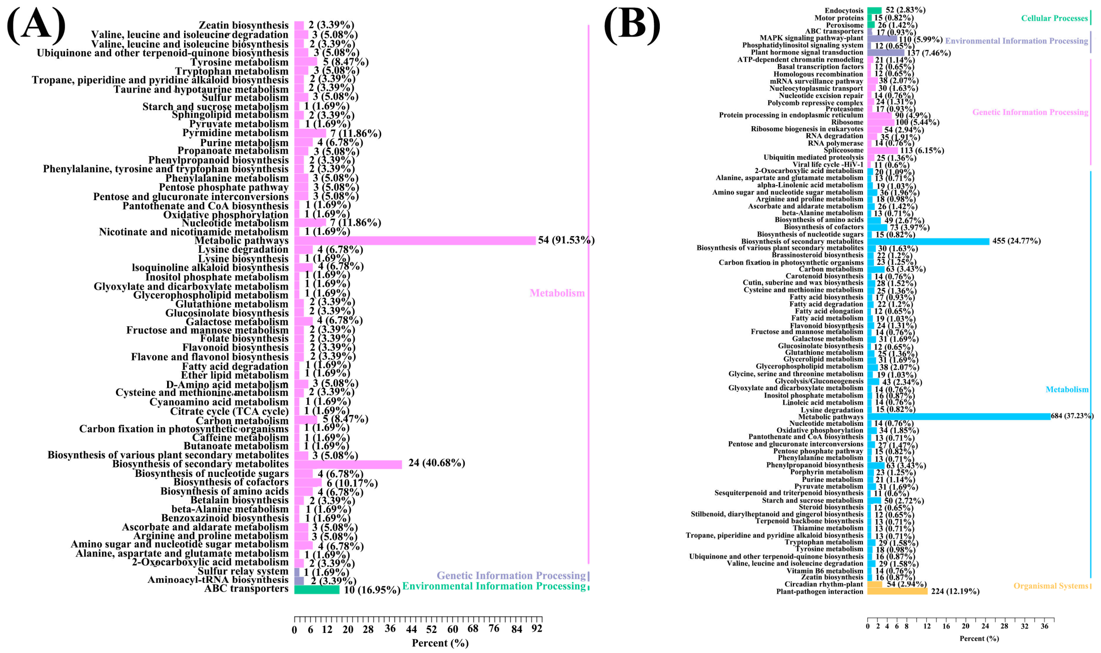

Figure S5. Metabolite and gene expression dynamics in the different quinoa samples. (A) Kyoto Encyclopedia of Genes and Genomes (KEGG) classification plot of the differentially expressed metabolites in sub classes 4 and 6. (B) KEGG classification plot of the differentially expressed genes in sub classes 5 and 8.

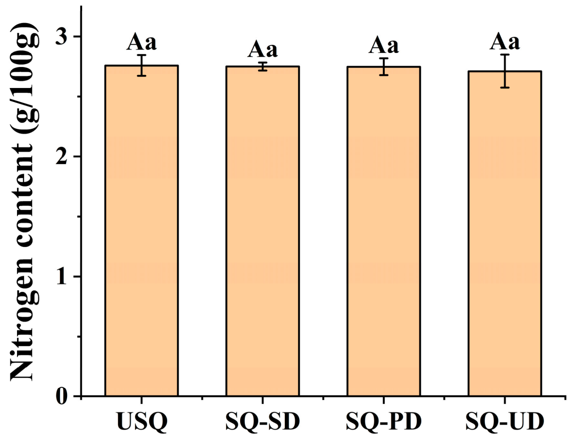

Figure S6. Differences in the levels of nitrogen content in the different quinoa samples. Different lowercase and capital letters indicate significant and extremely significant differences at $p < 0.05$ and $p < 0.01$, respectively.
